# The effectiveness and factors influencing frequency of endoscopic bougie dilatation in treating postoperative anastomotic stenosis of congenital esophageal atresia

**DOI:** 10.3389/fped.2024.1463165

**Published:** 2024-10-31

**Authors:** Xiao-qing He, Li-jing Xiong, Xiao-zhi Deng, Zheng-bing Yang, Huan Yan, Yi-xian Zhang, Li-hong Shang, Xiao-li Xie, Li-rong Liu, Jing Li

**Affiliations:** ^1^ Department of Pediatric Gastroenterology, Chengdu Women’s and Children’s Central Hospital, School of Medicine, University of Electronic Science and Technology of China, Chengdu, Sichuan, China; ^2^ Department of Pediatric Surgery, Chengdu Women’s and Children’s Central Hospital, School of Medicine, University of Electronic Science and Technology of China, Chengdu, Sichuan, China

**Keywords:** anastomotic stenosis, congenital esophageal atresia, endoscopic bougie dilatation, machine learning models, Random Forest model, logistic regression model, nomogram

## Abstract

**Objective:**

This study is to evaluate the effectiveness and frequency factors of endoscopic bougie dilatation in treating postoperative anastomotic stenosis of congenital esophageal atresia (CEA).

**Methods:**

The clinical data of patients of anastomotic stenosis with endoscopic bougie were retrospectively analyzed. According to the number of dilation times (ND), patients were divided into two groups (Group 0: ND < 3; Grooup1: ND ≥ 3), and the differences in multiple clinical data were compared. Lasso regression and Ridge regression were used to screen important variables. Classification models were built utilizing various machine learning algorithms and their performance were evaluated. Finally, Kaplan-Meier model was used to estimate the probable-time distribution of children achieving normal feeding.

**Results:**

Seventy-five patients underwent a total of 210 times of dilation, with a median of 3 times of dilation. The overall effectiveness was 98.67% (74/75), with perforation in 2 case (0.95%), and obvious bleeding in 3 cases (1.43%). Initial diameter of bougie, final diameter of bougie, treatment pattern (Regular: dilation each 4weeks; Wait-and-see: dilation until symptoms present), age at final dilation, esophageal obstruction by food were the factors related to ND. Random Forest (RF) and Logistic regression (LR) model were excellent models for predicting ND. The median age for achieving normal eating in Group0 was 120 days (95% CI: 90–160), while it was 270 days (95% CI: 240–460) in Group1 with a statistically significant difference (*P* < 0.0001).

**Conclusion:**

Endoscopic bougie dilatation is a safe and effective treatment for anastomotic stenosis. Selecting the appropriate bougie, using symptoms as the criterion for dilation, and minimizing the dilations under 3 times constitute a rational strategy.

## Introduction

The congenital esophageal atresia (CEA) is a common malformation of the esophagus in infants, which represents a severe condition within the spectrum of congenital malformations. The incidence of CEA ranges from 1/2,500 to 1/4,500 live births (1). CEA requires surgical correction, and postoperative survival rates have increased significantly due to improvements in surgical techniques and neonatal intensive care (2, 3). However, it is important to note that various complications may arise following surgery repair, with postoperative anastomotic stenosis being the most prevalent complication, occurring at an incidence rate ranging from 18% to 79% (4). Common predisposing factors for anastomotic stenosis included anastomotic suture material, tension at the anastomotic site, anastomotic leakage, prematurity, large esophageal pocket gap, and gastroesophageal reflux (5–12). The presence of anastomotic stenosis may result in postoperative dysphagia, vomiting, reflux. Severe anastomotic stenosis can lead to decreased oral intake, malnutrition, and the development of aspiration pneumonia.

Dilatation is required when postoperative anastomotic stenosis occurs. Balloon dilatation and bougie dilatation are two commonly employed methods in clinical practice (13). The comparison of the advantages and disadvantages of these two dilatation methods remains inconclusive, and the relevant clinical studies are mainly from adults. The available clinical studies on pediatric cohort are limited. However, a recent retrospective study demonstrated comparable safety, efficacy, and complication rates between the two methods. Considering the advantages of shorter operation time and cost-effectiveness, bougie dilatation appears to be more favorable than balloon dilatation (14), particularly in developing and under developed countries.

The objective of treating postoperative anastomotic stenosis with dilatation is to achieve an appropriate esophageal diameter that enables normal, age-appropriate feeding abilities without the manifestation of digestive or respiratory symptoms (15). Therefore, children often require repeated dilatation therapy, leading to an increased disease burden. To achieve the treatment goal while balancing the frequency of dilatations and symptom relief, it is essential to comprehend the factors influencing the number of dilatations. This retrospective study aimed to analyze the clinical data of patients who underwent endoscopic bougie dilatation for anastomotic stenosis after CEA repair, in order to assess the efficacy and safety of this procedure. Additionally, we investigated the factors influencing the frequency of dilatations and compared the duration taken for anastomotic stenosis children to achieve age-appropriate normal food intake, so as to provide a basis for formulating a more rational bougie dilatation strategy.

## Methods

### Patients

According to ESPGHAN-NASPGHAN (European Society for Pediatric Gastroenterology, Hepatology, and Nutritionand North American Society for Pediatric Gastroenterology, Hepatology, and Nutrition) Guidelines (15), postoperative anastomotic stenosis is defined as a stricture at the site of the esophageal anastomosis which can be detected by contrast radiography and/or endoscopy with significant functional impairment and symptoms. CEA is classified by Gross classification method (16).

#### Inclusion criteria

Infants diagnosed with CEA during the neonatal period and subsequently underwent surgical repair in our hospital, the tertiary children's hospitals in our region, during January 2013 to December 2022. After surgical CEA repair, the children exhibited clinical manifestations including vomiting, dysphagia, upper gastrointestinal reflux, feeding intolerance, and other related symptoms and anastomotic stenosis were confirmed by x-ray gastrointestinal contrast radiography. Meanwhile, the length and diameter of the stenosis were measured. Then, the endoscopic dilatation procedure with bougie were performed on all children diagnosed with anastomotic stenosis.

#### Exclusion criteria

Other dilatation methods were used during the treatment duration, such as balloon dilatation, dilatation under x-ray radiography, endoscopic stenotomy, and gastrostomy; combined with other causes of esophageal stenosis, such as esophageal corrosions, esophageal foreign bodies injury, eosinophilic esophagitis; children with anastomotic leakage or esophageal fistula, severe esophageal inflammation, and severe cardiopulmonary comorbidities; long-term follow-up data were not available.

The procedure of this study adhered to the ethical standards established by the Human Trial Committee of Chengdu Women and Children's Central Hospital and received approval from the hospital's ethics committee. Simultaneously, informed consent was obtained from all parents of the participants.

#### Endoscopic bougie dilatation

The electronic gastroscope used was the EG-250PE model from Fujinon Company (Japan). A JHK type esophageal stricture dilator (Changzhou Jiuhong Medical Equipment Co., LTD, China.) was utilized, along with a complete set of dilators consisting of 5 expansion bars and 1 guide wire. The expansion bougie diameters ranged from 5.0 mm to 13.0 mm, each measuring at a length of 90.0 cm. The guide wire had a diameter of 2.0 mm and featured a spring at its front end. All the dilatation procedure were performed under endoscope. The preoperative examination was conducted to rule out any contraindications for anesthesia and surgery, and the procedures were conducted under general anesthesia with endotracheal intubation. Firstly, the gastroscope was utilized for the identification of esophageal stricture. The dimensions of the stricture and the distance from incisor to incisor were assessed. The remnant of the esophageal cavity was aspirated, followed by insertion of a metallic guide wire through the stricture site via the endoscopic biopsy hole, and subsequent withdrawal of the gastroscope while retaining the guide wire.

The dilation procedure is carried out by physicians who possess extensive experience and maintain a consistent level of expertise. The bougie with an appropriate diameter was selected based on the results of endoscopic evaluation and preoperative gastrointestinal contrast radiography, and it is guided along the wire to pass through the stenosis site. When the operator felt resistance, continue to slowly push the bougie to reach the distal end of the stenosis or make the front end of the expander reach the front spring of the guide wire. After maintaining the dilatation operation for a duration of 3–5 min, the bougie was withdrawn and the position of the guide wire was kept relatively stable. The same procedure was then sequentially performed using bougie of larger diameter. The dilatation was halted until the operator encountered significant resistance and observed a small amount of blood at the distal end of the exit bougie. After the completion of the expansion, the dilatation bougie was withdrawn simultaneously with the guide wire. Finally, the gastroscope was inserted into the distal end of the dilated stricture again, and the degree of esophageal dilatation, bleeding and complications were observed.

#### Study design

Patients completed dilatation therapy and were divided into two groups based on the number of dilatation times (Group 0: ND < 3 dilatation times; Group 1: ND ≥ 3 dilatation times). The two groups were compared in terms of gender, gestational age, birth weight (BW), type of CEA, associated malformations (AM), initial weight for dilatation (IWD), time of detecting anastomotic stenosis (Tanastomotic stenosis), interval between CEA surgical repair and first dilation (ISD), onset age of dilation (OAD), initial diameter of bougie (IDB) (5 mm, 7 mm, 9 mm, 11 mm), final diameter of bougie (FDB) (7 mm, 9 mm, 11 mm, 13 mm, 15 mm), complications after dilation, Initial diameter of anastomotic stenosis (IDanastomotic stenosis), treatment pattern (TP) (Regular: dilation each 3–4weeks; Wait-and-see: dilation until symptoms present) in order to study the related factors to the frequency of dilatations. Meanwhile, the age at final dilation (AFD), esophageal obstruction (EO) by food, age of regular intake (ARI), and interval between final dilation and regular intake (IFDRI) were compared between groups. Furthermore, the comparison was also made of duration in which children with different dilation times to reach the age-appropriate normal eating.

#### Follow-up visit

The children who underwent dilatation treatment were followed up for a duration of 12 months after the completion of the last bougie dilatation procedure. If they did not achieve the age-appropriate dietary intake by the end of this follow-up period, further monitoring was continued until they achieved the normal food intake, and the duration was recorded.

### Statistical analysis

All Statistical analyses were performed using R version 4.2.3 and python version 3.11.4. Categorical data were presented as numerical values and percentages, while normally distributed continuous variables were expressed as means ± standard deviations (SD). Non-normally distributed variables were represented by medians and interquartile ranges (IQR). The comparison between the two groups for categorical variables was conducted using the χ^2^ test or Fisher's exact test. For continuous variables that deviated from normal distribution, the Mann-Whitney-U nonparametric test was employed for comparison. Univariate analysis was performed using a binary logistic regression model. Variable selection (binomial) was carried out using Lasso regression model, followed by ranking of filtered variables based on their importance (using Ridge regression). Classification models were built utilizing various machine learning algorithms including XGB Classifier, Logistic Regression, Random Forest Classifier, MLP Classifier, Ada Boost Classifier, K-Neighbors Classifier, Gaussian NB, SVC and Gradient Boosting Classifier. The two-fold cross method was used for verification, and the indicators of the training set and the validation set were compared including AUC (Area Under Curve), specificity, sensitivity, accuracy, positive predictive value (PPV) and negative predictive value(NPV) between the training set and validation set. The optimal algorithm was selected to construct the final prediction model regarding the frequency of dilation. Finally, The Kaplan-Meier model was used to estimate the probable-time distribution of children reaching normal feeding in the two groups, and the Log-rank test was used to assess the difference. *P* < 0.05, the difference was statistically significant.

## Results

### Demographic and clinical characteristics

A total of 80 children were diagnosed with anastomotic stenosis after CEA, including 41 males (51.25%) and 39 females (48.75%). The age of the initial endoscopic bougie dilatation ranged from 31 to 474 days with the median age of 68.000 [47.000, 104.000] days. The CEA types were all classified as type Ⅲ, with type Ⅲa presented in 52 cases (65%) and type Ⅲb presented in 28 cases (35%). The patients all underwent CEA surgical repair during the neonatal period, and contrast radiography was conducted at 4 weeks post-surgery to evaluate the presence of anastomotic stenosis. Throughout the treatment period, three children were relinquished by their family members, and two children underwent gastrostomy due to severe anastomosis stenosis during the second expansion. The remaining 75 patients with anastomotic stenosis had at least one symptom of feeding problems including vomiting (70/75, 99.33%), reflux (45/75, 60%), and poor intake (35/75, 46.67%).

### Efficacy of endoscopic bougie dilatation

The remaining 75 cases underwent a total of 210 times of dilation, ranging from 1 to 13 times, with a median of 3 times of dilation. The patients were categorized into Group 0 (ND < 3) and Group 1 (ND ≥ 3) based on the frequency of dilation. Perforation occurred in 2 case (2/210, 0.95%), and obvious bleeding occurred in 3 cases (1.43%), all of which recovered after treatment with short fasting. Seventy-four children finally reached the normal food intake corresponding to their age. Only one patient still had recurrent vomiting at the end of follow-up and did not continue dilatation. Therefore, the overall effective rate of expansion treatment was 98.67% (74/75).

### Comparison of related factors affecting the frequency of dilation

The age at the last dilation differed significantly between the two groups (*P* < 0.001). When the number of dilations exceeded 3, there was a statistically significant difference in the older age at the end of dilation. The diameter of the final expansion showed a significant difference between the two groups (*P* = 0.002). In the group with less dilation, 7 mm accounted for 40%, 9 mm accounted for 29%, and 11 mm accounted for 31% of the diameter of bougie, indicating a relatively balanced distribution, with no bougie of 13 mm and 15 mm observed. Among those who underwent more than three times of dilation, majority had a final diameter of 11 mm, accounting for 53%. There was a significant difference in dilatation strategy between the two groups (*P* < 0.001) as shown in Table 1. In the group with fewer dilatation, most children (71%) adopted a wait-and-see strategy where re-dilatation occurred when symptoms appeared. Conversely, regular dilation resulted in more times in 80% of patients. Additionally, univariate logistic regression analysis revealed that apart from AFD, FDB and TP factors, esophageal obstruction (EO) was also identified as a risk factor for undergoing more than three dilatations (*P* = 0.048) as shown in Table 2.

**Table 1:** Demographic and factors between groups with different number of dilation times.

Variable	Number of Dilation times <3 times (*N* = 35)	Number of Dilation times ≥3 times (*N* = 40)	*p*-value
Gender, *n* (%)			0.877
0	19 (54)	21 (53)	
1	16 (46)	19 (48)	
BW (kg), Median (IQR)	3.000 (2.575–3.275)	2.775 (2.400–3.000)	0.164
IDAS (mm), Median (IQR)	4.000 (3.000–5.000)	3.000 (2.000–4.250)	0.100
IWD (kg), Median (IQR)	4.500 (3.550–5.200)	4.000 (3.500–5.000)	0.307
TAS (days), Median (IQR)	60.000 (38.000–84.000)	46.500 (35.000–77.250)	0.466
ISD (days), Median (IQR)	82.000 (47.000–114.000)	52.500 (44.750–97.250)	0.258
OAD (days), Median (IQR)	84.000 (50.500–115.500)	54.500 (47.000–99.250)	0.232
AFD (days), Median (IQR)	101.000 (63.000–158.000)	278.000 (211.000–515.000)	<0.001
Preterm, *n* (%)			0.384
No	27 (77)	34 (85)	
Yes	8 (23)	6 (15)	
CEA, *n* (%)			0.870
Ⅲa	23 (66)	27 (68)	
Ⅲb	12 (34)	13 (33)	
AM, *n* (%)			0.558
No	19 (54)	19 (48)	
Yes	16 (46)	21 (53)	
IDB, *n* (%)			0.058
5 mm	18 (51)	31 (78)	
7 mm	11 (31)	7 (18)	
9 mm	5 (14)	1 (2.5)	
11 mm	1 (2.9)	1 (2.5)	
FDB, *n* (%)			0.002
7 mm	14 (40)	3 (7.5)	
9 mm	10 (29)	11 (28)	
11 mm	11 (31)	21 (53)	
13 mm	0 (0)	4 (10)	
15 mm	0 (0)	1 (2.5)	
Complications, *n* (%)			0.596
No	33 (94)	39 (98)	
Yes	2 (5.7)	1 (2.5)	
TP, *n* (%)			<0.001
Wait-and-see*a	25 (71)	8 (20)	
Regular dilation*b	10 (29)	32 (80)	
EO, *n* (%)			0.113
No	34 (97)	34 (85)	
Yes	1 (2.9)	6 (15)

**Table 2:** Univariate logistic regression of factor related to the dilation times.

Factors	*P*	Odds ratio	Lower	Upper
BW	0.191	0.3	0.036	1.599
IWD	0.206	0.556	0.192	1.213
IDES	0.537	0.808	0.395	1.616
AFD	0.037	1.014	1.003	1.029
IDB (7 mm)	0.385	0.354	0.027	3.719
IDB (9 mm)	0.105	0.053	0.001	1.411
FDB (9 mm)	0.025	23.713	1.948	602.727
FDB (11 mm)	0.081	13.084	0.899	359.624
TP	0.003	56.628	6.054	1,457.909
EO	0.048	64.936	1.469	9,266.423

**Figure 1:**
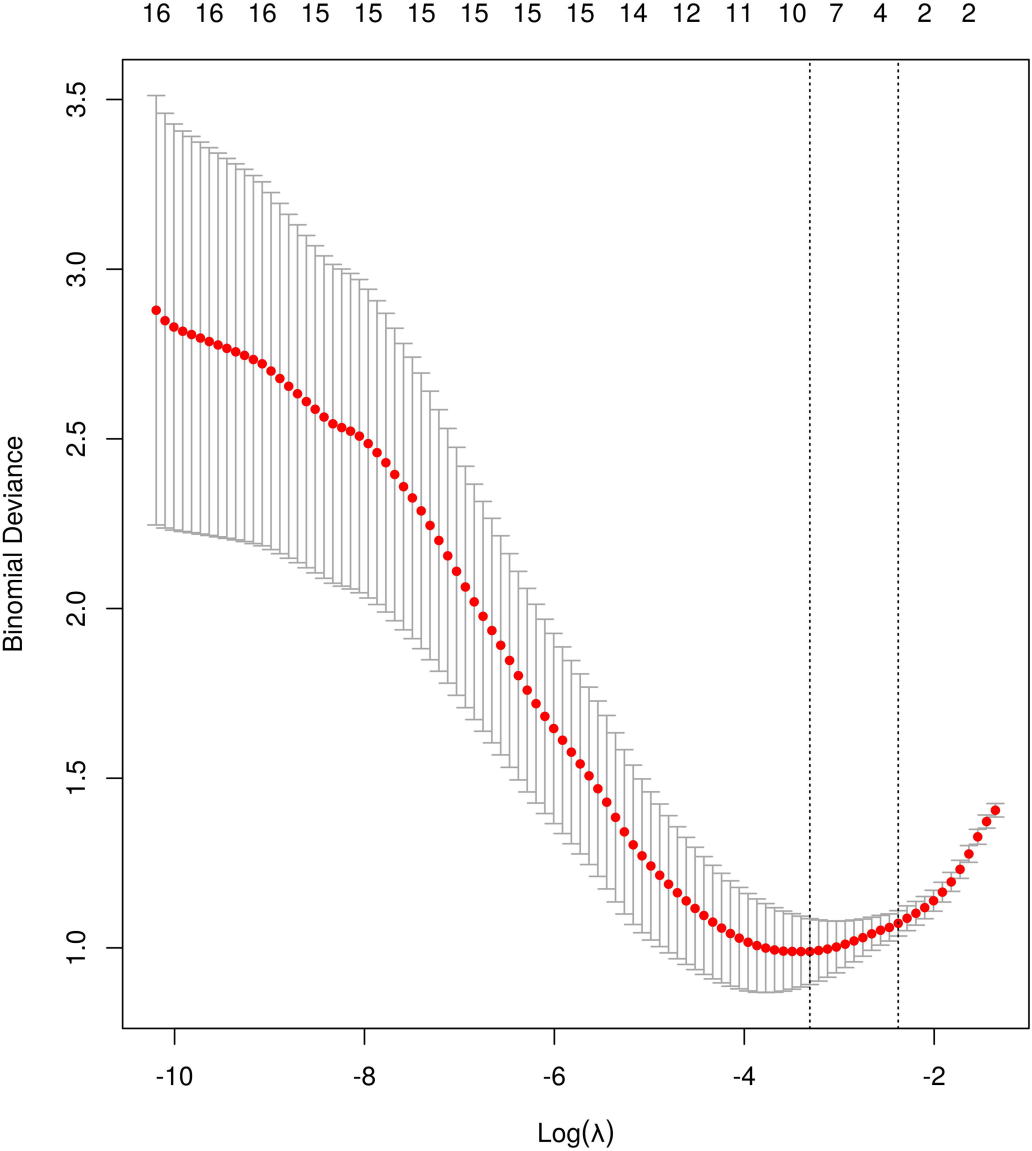
Coefficients of the LASSO model: LASSO coefficient profiles of the 4 features. A coefficient profile plot was produced against the log λ sequence (0.093).

**Figure 2:**
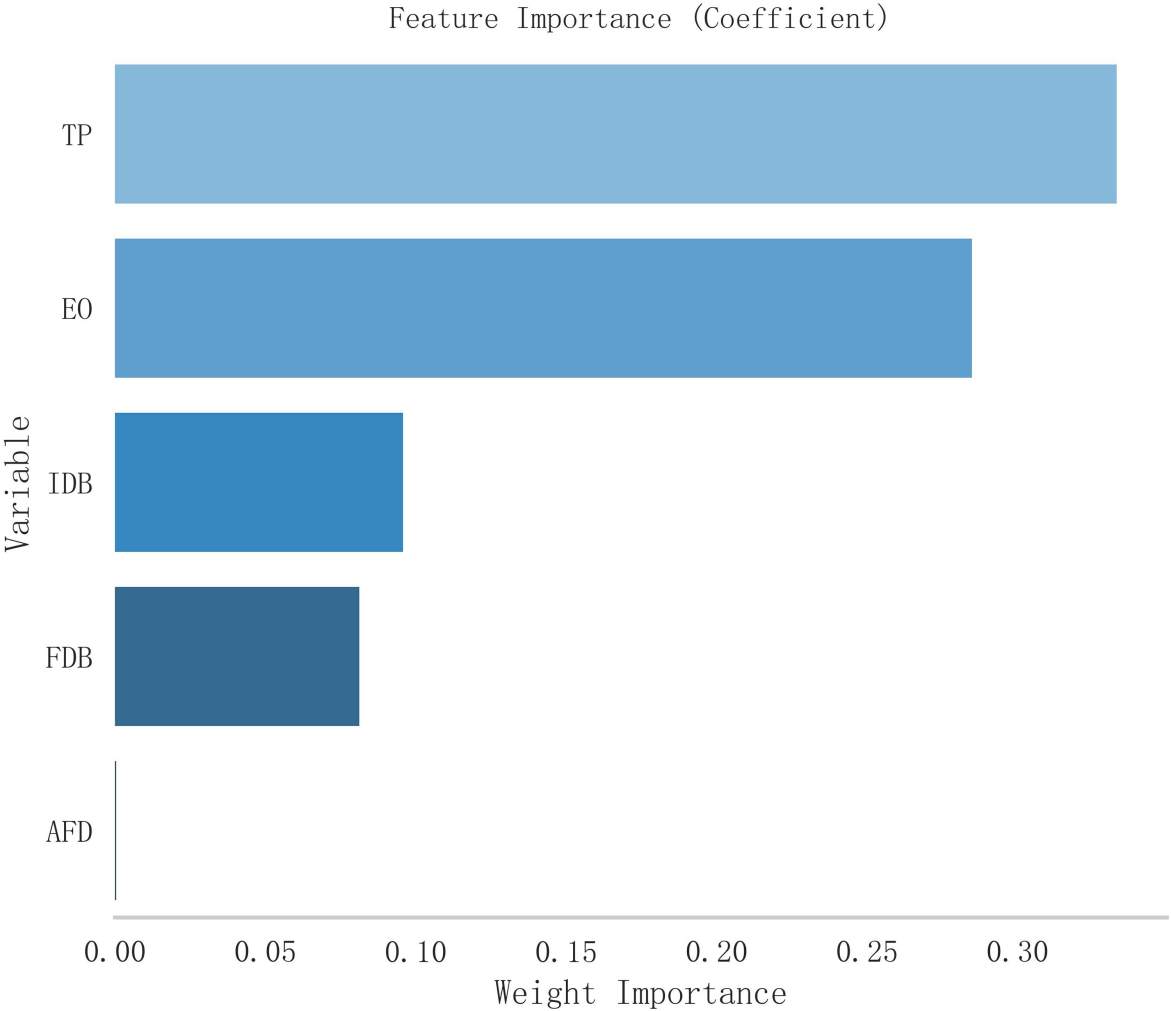
Coefficients of importance. TP, treatment pattern (0.333); EO, esophageal obstruction (0.285); IDB, initial diameter of bougie (0.096); FDB, final diameter of bougie (0.081); AFD, age at final dilation (0.000).

The variable selection process employed Lanastomotic stenosisSO regression analysis (Figure 1), with a λ value of 0.093 based on the standard error of the minimum distance. The selected variables in the model were IDB, FDB, TP, and AFD. Subsequently, Ridge regression was utilized to rank their feature importance, resulting in the following order from high to low: TP, EO, IDB, FDB, AFD (Figure 2). Consequently, TP was considered as the most influential factor.

**Figure 3:**
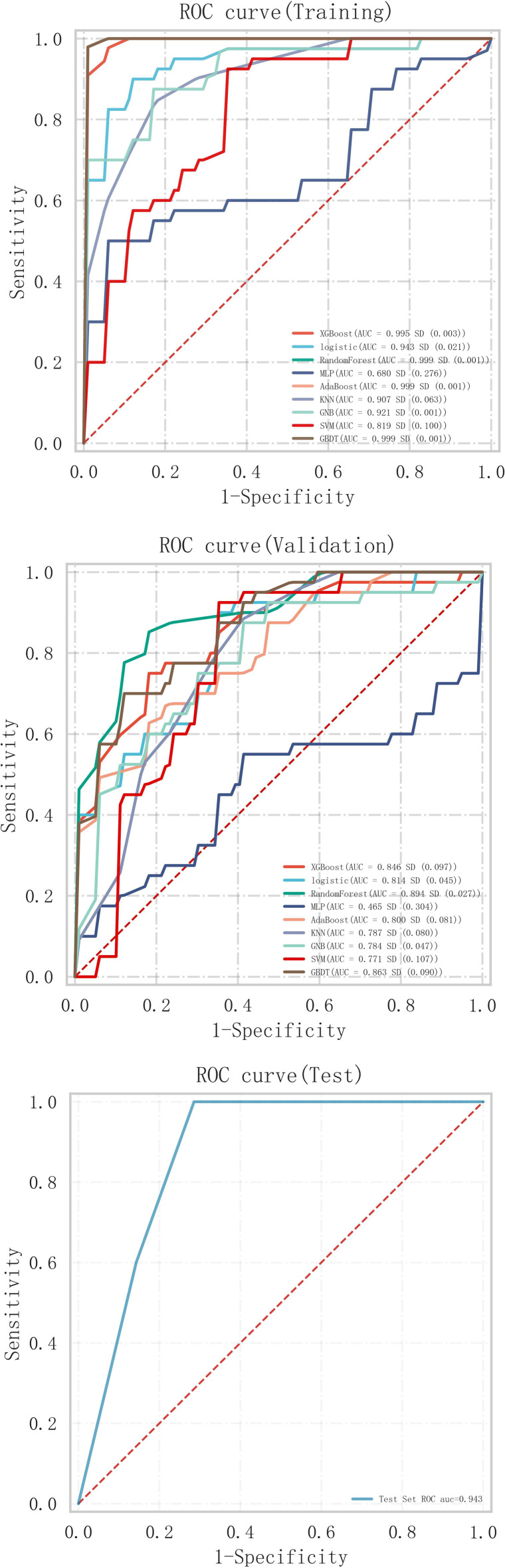
**(A)** Predicted ROC curve of training sets of machine learning models. **(B)** Predicted ROC curve of validating sets of machine learning models. **(C)** Predicted ROC curve of Testing sets of machine learning models. MLP, multilayer perceptron; KNN, K-nearest neighbor; GNB, Gaussian Naïve Bayes; SVM, support vector machines; GBDTA, gradient boosting decision tree.

The selected variables are incorporated into various machine learning models including XGB Classifier, Logistic Regression (LR), Random Forest Classifier (RF), MLP Classifier, Ada Boost Classifier, K-Neighbors Classifier, Gaussian NB, SVC, and Gradient Boosting Classifier. Among these models evaluated on the training set (as shown in Tables 3, 4), the Random Forest model along with the AdaBoost model and GBDT demonstrated superior performance (Figure 3A). However, when tested on validation set, the Random Forest model outperformed all other models (Figure 3B). Consequently, we have chosen to utilize the Random Forest model as our final selection. To assess its discriminative power accurately, we conducted a detailed analysis of this individual RF model which yielded specificity of 78.6%, sensitivity of 91.3%, accuracy of 77.8%, and an AUC value of 0.943 (Figure 3C). The pkl file for machine learning by RF model was provided in the Supplementary Material.

**Table 3:** Evaluation metrics for the training dataset in different model.

Model	AUC (SD)	Accuracy (SD)	Sensitivity (SD)	Specificity (SD)	PPV (SD)	NPV (SD)	F1 score (SD)	Kappa (SD)
XGBoost	0.995 (0.003)	0.933 (00.012)	0.950 (00.000)	0.972 (00.028)	0.974 (00.026)	0.895 (00.000)	0.962 (00.013)	0.867 (00.025)
LR	0.943 (00.021)	0.866 (00.028)	0.825 (00.075)	0.972 (00.028)	0.972 (00.028)	0.795 (00.055)	0.889 (00.032)	0.736 (00.054)
RF	0.999 (00.001)	0.960 (00.014)	0.975 (00.025)	1.000 (00.000)	1.000 (00.000)	0.921 (00.026)	0.987 (00.013)	0.920 (00.028)
MLP	0.680 (00.276)	0.677 (00.191)	0.500 (00.350)	0.943 (00.002)	0.804 (00.137)	0.640 (00.169)	0.569 (00.324)	0.388 (00.350)
AdaBoost	0.999 (00.001)	0.960 (00.014)	0.975 (00.025)	1.000 (00.000)	1.000 (00.000)	0.921 (00.026)	0.987 (00.013)	0.920 (00.028)
KNN	0.907 (00.063)	0.746 (00.043)	0.800 (00.050)	0.884 (00.060)	0.923 (00.077)	0.659 (00.034)	0.857 (00.062)	0.503 (00.084)
GNB	0.921 (00.001)	0.853 (00.011)	0.850 (00.050)	0.917 (00.083)	0.925 (00.075)	0.803 (00.030)	0.882 (00.007)	0.709 (00.025)
SVM	0.819 (00.100)	0.786 (00.056)	0.850 (00.000)	0.768 (00.121)	0.808 (00.081)	0.767 (00.033)	0.826 (00.043)	0.568 (00.117)
GBDT	0.999 (00.001)	0.960 (00.014)	0.975 (00.025)	1.000 (00.000)	1.000 (00.000)	0.921 (00.026)	0.987 (00.013)	0.920 (00.028)

**Table 4:** Evaluation metrics for the validation set in different model.

Model	AUC (SD)	Accuracy (SD)	Sensitivity (SD)	Specificity (SD)	PPV (SD)	NPV (SD)	F1 score (SD)	Kappa (SD)
XGBoost	0.846 (0.097)	0.732 (0.110)	0.725 (0.125)	0.856 (0.033)	0.802 (0.135)	0.676 (0.097)	0.762 (0.130)	0.466 (0.221)
LR	0.814 (0.045)	0.773 (0.016)	0.825 (0.125)	0.796 (0.149)	0.834 (0.095)	0.747 (0.039)	0.815 (0.017)	0.544 (0.040)
RF	0.894 (0.027)	0.773 (0.016)	0.850 (0.050)	0.856 (0.033)	0.876 (0.053)	0.704 (0.004)	0.863 (0.051)	0.551 (0.034)
MLP	0.465 (0.304)	0.549 (0.181)	0.475 (0.475)	0.794 (0.206)	0.514 (0.214)	0.563 (0.170)	0.412 (0.412)	0.109 (0.342)
AdaBoost	0.800 (0.081)	0.692 (0.097)	0.900 (0.100)	0.678 (0.266)	0.788 (0.141)	0.629 (0.079)	0.823 (0.037)	0.390 (0.195)
KNN	0.787 (0.080)	0.573 (0.032)	0.800 (0.000)	0.711 (0.123)	0.707 (0.071)	0.526 (0.026)	0.749 (0.040)	0.171 (0.061)
GNB	0.784 (0.047)	0.732 (0.084)	0.850 (0.100)	0.709 (0.180)	0.775 (0.108)	0.693 (0.068)	0.797 (0.014)	0.462 (0.172)
SVM	0.771 (0.107)	0.745 (0.123)	0.850 (0.000)	0.768 (0.121)	0.781 (0.114)	0.711 (0.132)	0.810 (0.062)	0.491 (0.246)
GBDT	0.863 (0.090)	0.719 (0.044)	0.900 (0.000)	0.739 (0.150)	0.875 (0.125)	0.643 (0.024)	0.883 (0.065)	0.447 (0.089)

**Figure 4:**
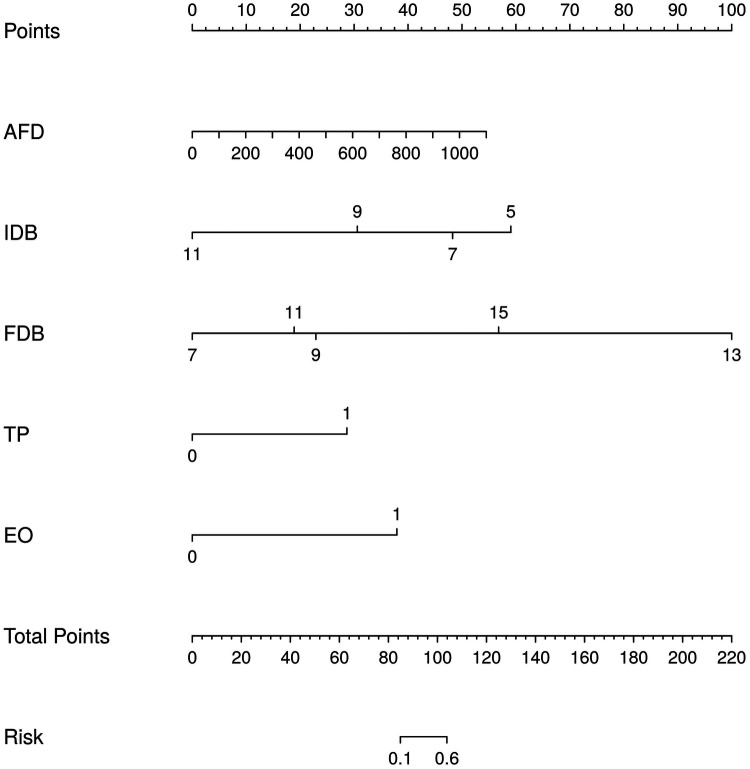
Nomogram of LR model of dilation times. The probability for an individual patient is estimated by acquiring the value of each factor on each variable axis, drawing a line upwards to determine the point, locating the sum of these numbers on the total point axis, and drawing a line downwards to determine the likelihood of an adverse event on the risk axis. AFD, age at final dilation; IDB, initial diameter of bougie; FDB, final diameter of bougie; TP, treatment pattern; EO, esophageal obstruction.

Finally, the logistic regression (LR) model, being the most commonly employed predictive model in clinical practice, presents variables as nomograms following their inclusion (Figure 4). Model calibration was evaluated using the Hosmer-Lemeshow test, revealing no significant disparity between observed and expected values (*P* = 0.979). Furthermore, the model exhibited excellent overall performance with a Brier Score of 0.084 on the calibration curve.

### Comparison of the time to normal feeding in children with different dilation times

**Figure 5:**
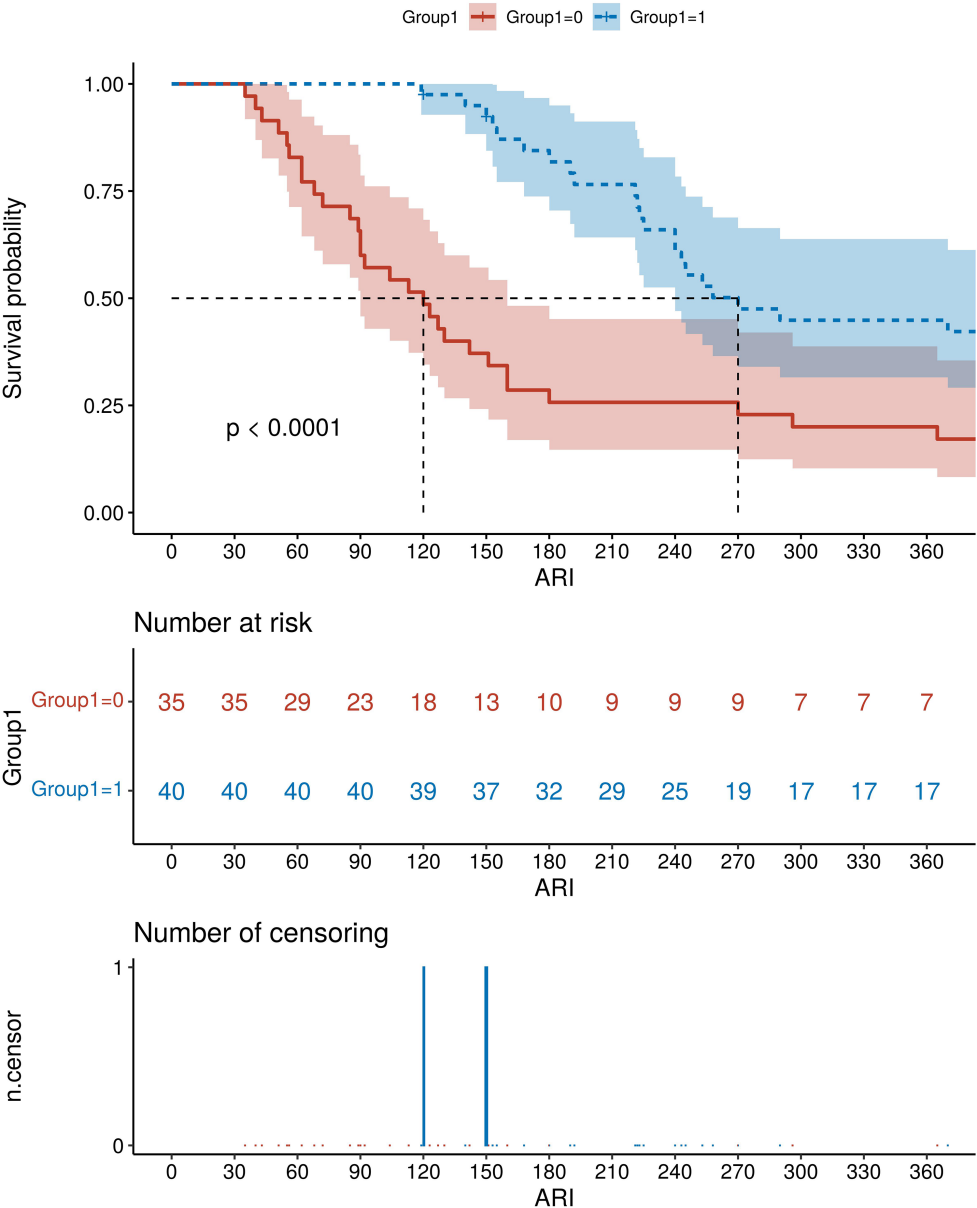
The cumulative probability of AS children with different dilation times (group 0: dilation time <3 times; Group1: dilation times ≥3 times) to achieve the normal eating. ARI, age of regular intake.

The children were followed up for a period of 12 months after the final dilatation, and it was found that 72 children (96%) had achieved a normal feeding. However, two children did not eat normally, and the follow-up time was extended to 630 days and 462 days after final dilatation, respectively. Eventually, both of them reached a level of normal eating corresponding to their age. The median age for achieving age-appropriate normal eating in Group 0 was 120 days (95% CI: 90–160). In the group with 3 or more dilatation times, the median age for achieving age-appropriate normal eating was 270 days (95% CI: 240–460). The Log-rank test statistic between the two groups yielded a value of 17.103, HR = 0.39995%, 95% CI: 0.236–0.675, *P* < 0.0001, indicating a statistically significant difference (Figure 5).

## Discussion

The occurrence of anastomotic stenosis is a prevalent complication following CEA repair. Currently, the primary treatment for anastomotic stenosis after CEA involves esophageal dilatation, with guidelines recommending the utilization of guide-wire dilatation therapy encompassing balloon and bougie dilatation (15). The safety of these two kinds of dilatation has been previously reported, and an equivalence study was conducted to assess their effectiveness (13, 17, 18). In this study, a total of 75 children successfully completed dilatation using endoscopic bougie dilatation. The overall effectiveness rate was 98.67%, with only two instances of perforation, resulting in a low perforation rate (0.95%), indicating excellent safety. The effectiveness rate of this study exceeded the previously reported success rate (80.4%) of balloon dilatation in treating anastomotic stenosis after CEA (19). Furthermore, it is also higher than those in two previous studies of bougie dilation (87%–90%) (8, 20). The reason could be speculated, possibly associated with anastomotic stenosis as the single disease in all subjects included in this study. Thus, this implied that endoscopic bougie dilatation was an effective approach for treating anastomotic stenosis children after CEA surgery.

The ultimate goal of dilation in children with anastomotic stenosis is to achieve an optimal esophageal diameter that enables age-appropriate oral feeding ability without any digestive or respiratory symptoms (15). The repeated dilatation in clinical treatment not only escalates the risks of anesthesia and infection, but also amplifies the financial burden on the family. First of all, we found that approximately half of the patients with anastomotic stenosis (46.67%) were able to eat normally with less than three dilation times, with a diameter ≤11 mm. Therefore, we conducted a comparative analysis of various clinical factors between the two groups by categorizing them based on the criterion of three times of dilation treatment. Statistical analysis showed that the diameter of the bougie, age, symptoms (whether food was obstructed in the esophagus during the treatment duration) and the strategy of dilatation were the main factors affecting the times of dilatation. The smaller the initial bougie diameter, the greater the number of dilations required at the end. Meanwhile, a larger final bougie diameter necessitates more frequent dilations. Moreover, achieving normal eating through regular dilatation requires a significantly higher total number of dilations compared to symptomatic dilatation. The initial selection of smaller diameter bougie, of course, suggested a more severe degree of esophageal stenosis.

The study conducted by Park JY and Park JM et al. (21) demonstrated that if the number of dilations exceeded three times without significant improvement in symptoms, further increasing the times of dilation did not yield a statistically significant enhancement in treatment efficacy. The machine learning algorithm exhibits evident advantages over traditional statistics in terms of model flexibility, processing large-scale data, variable handling, prediction accuracy, and application domains. Therefore, based on clinical data and rational variable selection methods, we used multiple machine learning (ML) algorithms to try to establish a model that could predict the number of dilations more than 3 times. The RF model was chosen as the optimal model after assessing its performance on both the training and validation sets. We provided the pkl file as Supplementary Material. Meanwhile, we also demonstrated the nomogram of LR which was the most commonly used model in the clinical practice. It was expected that future researchers could use clinical data from multiple centers to verify the model, so as to calibrate and improve the model, and provide more evaluation methods for clinical treatment.

The cumulative probability of time to achieve normal eating differed significantly among children with varying dilatation times. The median time to achieve a normal diet was extended by 5 months in children with dilation exceeding 3 times compared to those with dilation below 3 times.

Therefore, those results suggested that the goal of dilatation therapy should focus on achieving symptom relief rather than pursuing an increased esophageal diameter, in order to minimize the number of dilations for a maximum of three times. This was consistent with the results of multi-center studies in other country (22).

In conclusion, endoscopic bougie dilatation is a safe and effective treatment for anastomotic stenosis after CEA repair surgery. Selecting the appropriate bougie diameter, using symptoms as the criterion for repeated dilation, and minimizing the number of dilations to less than 3 times constitute a rational strategy for treatment. Additionally, we employed RF algorithm to develop a prediction model for cases requiring more than 3 dilatations, aiming to provide assistance in future efficacy predictions. However, it should be noted that this study has limitations as it is a single-center retrospective data analysis with a small sample size and utilizes relatively basic clinical indicators as analysis factors. We anticipate validation and improvement through future multi-center studies along with the identification of additional markers associated with dilation effects in order to construct more comprehensive treatment strategies.

## Data Availability

The original contributions presented in the study are included in the article/Supplementary Material, further inquiries can be directed to the corresponding author.

## References

[1] Pinheiro PF Simões e Silva AC Pereira RM. Current knowledge on esophageal atresia. World J Gastroenterol. (2012) 18(28):3662–72. 10.3748/wjg.v18.i28.3662 22851858 PMC3406418

[2] Chiang CM Hsu WM Chang MH Hsu HY Ni YH Chen HL Risk factors and management for anastomotic stricture after surgical reconstruction of esophageal atresia. J Formos Med Assoc. (2021) 120(1 Pt 2):404–10. 10.1016/j.jfma.2020.06.020 32586720

[3] Konkin DE O’Hali WA Webber EM Blair GK. Outcomes inesophageal atresia and tracheoesophageal fistula. J Pediatr Surg. (2003) 38:1726–9. 10.1016/j.jpedsurg.2003.08.039 14666453

[4] Boeder AN Lal DR. Advances in the surgical management of esophageal atresia. Adv Pediatr. (2021) 68:245–59. 10.1016/j.yapd.2021.05.004 34243856

[5] Chittmittrapap S Spitz L Kiely EM Brereton RJ. Anastomotic leakage following surgery for esophageal atresia. J Pediatr Surg. (1992) 27:29–32. 10.1016/0022-3468(92)90098-R 1552439

[6] Touloukian RJ Seashore JH. Thirty-five-year institutional experience with end-to-side repair for esophageal atresia. Arch Surg. (2004) 139:371–4. discussion 4. 10.1001/archsurg.139.4.371 15078702

[7] Chittmittrapap S Spitz L Kiely EM Brereton RJ. Anastomotic stricture following repair of esophageal atresia. J Pediatr Surg. (1990) 25:508–11. 10.1016/0022-3468(90)90561-M 2352084

[8] Serhal L Gottrand F Sfeir R Guimber D Devos P Bonnevalle M Anastomotic stricture after surgical repair of esophageal atresia: frequency, risk factors, and efficacy of esophageal bougie dilatations. J Pediatr Surg. (2010) 45:1459–62. 10.1016/j.jpedsurg.2009.11.002 20638524

[9] Upadhyaya VD Gangopadhyaya AN Gupta DK Sharma SP Kumar V Pandey A Prognosis of congenital tracheoesophageal fistula with esophageal atresia on the basis of gap length. Pediatr Surg Int. (2007) 23(8):767–71. 10.1007/s00383-007-1964-0 17579871

[10] Shah R Varjavandi V Krishnan U. Predictive factors for complications in children with esophageal atresia and tracheoesophageal fistula. Dis Esophagus. (2015) 28:216–23. 10.1111/dote.12177 24456536

[11] Antoniou D Soutis M Christopoulos-Geroulanos G. Anastomotic strictures following esophageal atresia repair: a 20-year experience with endoscopic balloon dilatation. J Pediatr Gastroenterol Nutr. (2010) 51:464–7. 10.1097/MPG.0b013e3181d682ac 20562719

[12] Parolini F Leva E Morandi A Macchini F Gentilino V Di Cesare A Anastomotic strictures and endoscopic dilatations following esophageal atresia repair. Pediatr Surg Int. (2013) 29:601–5. 10.1007/s00383-013-3298-4 23519549

[13] Ghiselli A Bizzarri B Ferrari D Manzali E Gaiani F Fornaroli F Endoscopic dilatation in pediatric esophageal strictures: a literature review. Acta Biomed. (2018) 89(8-S):27–32. 10.23750/abm.v89i8-S.7862 30561414 PMC6502217

[14] Fakıoglu E Güney LH Ötgün İ. Esophageal dilatation through bouginage or balloon catheters in children, as the treatment of benign esophageal strictures: results, considering the etiology, and the methods. Çocuklarda benign özofagus darlıklarının tedavisinde bujinaj veya balonla özofagus dilatasyonu: etiyoloji ve yöntemlere göre sonuçlar. Ulus Travma Acil Cerrahi Derg. (2023) 29(5):574–81. 10.14744/tjtes.2022.03881 37145049 PMC10277326

[15] Krishnan U Mousa H Dall'Oglio L Homaira N Rosen R Faure C ESPGHAN-NASPGHAN guidelines for the evaluation and treatment of gastrointestinal and nutritional complications in children with esophageal atresia-tracheoesophageal Fistula. J Pediatr Gastroenterol Nutr. (2016) 63(5):550–70. 10.1097/MPG.0000000000001401 27579697

[16] Harmon CM Coran AG. Congenital anomalies of the esophagus. In: Coran AG Adzick NS Krummel TM Laberge JM Shamberger RC Caldamone AA, editors. Pediatric Surgery. 7th ed. Philadelphia: Mosby Elsevier (2012). p. 893–918.

[17] Manfredi MA. Endoscopic management of anastomotic esophageal strictures secondary to esophageal atresia. Gastrointest Endosc Clin N Am. (2016) 26:201–19. 10.1016/j.giec.2015.09.002 26616905

[18] George A Sinha V. Balloon and bougie dilatation of benign esophageal strictures. Indian J Otolaryngol Head Neck Surg. (2005) 57:196–8. 10.1007/BF03008012 23120170 PMC3451343

[19] Raboei E Alabdali A Sayed MH Yousef Y Bawazir O Alsaggaf A The outcome of pediatric esophageal strictures managed with endoscopic balloon dilation in Saudi Arabia. J Laparoendosc Adv Surg Tech A. (2021) 31(2):210–5. 10.1089/lap.2020.0455 33216676

[20] Michaud L Guimber D Sfeir R Rakza T Bajja H Bonnevalle M Sténose anastomotique après traitement chirurgical de l'atrésie de l'oesophage: fréquence, facteurs de risque et efficacité des dilatations oesophagiennes [Anastomotic stenosis after surgical treatment of esophageal atresia: frequency, risk factors and effectiveness of esophageal dilatations]. Arch Pediatr. (2001) 8(3):268–74. (In French). 10.1016/S0929-693X(00)00193-7 11270250

[21] Park JY Park JM Shin GY Kim JS Cho YK Kim TH Efficacy of bougie dilatation for normal diet in benign esophageal stricture. Scand J Gastroenterol. (2023) 58(2):199–207. 10.1080/00365521.2022.2111227 35996943

[22] Koivusalo A Pakarinen MP Rintala RJ. Anastomotic dilatation after repair of esophageal atresia with distal fistula. Comparison of results after routine versus selective dilatation. Dis Esophagus. (2009) 22(2):190–4. 10.1111/j.1442-2050.2008.00902.x 19207547

